# Analysis of predicted B and T-cell epitopes in Der p 23, allergen from Dermatophagoides pteronyssinus

**DOI:** 10.6026/97320630013307

**Published:** 2017-09-30

**Authors:** Songwe Fanuel, Saeideh Tabesh, Esmaeil Sadroddiny, Gholam Ali Kardar

**Affiliations:** 1 Department of Medical Biotechnology, School of Advanced Technologies in Medicine, Tehran University of Medical Sciences-International Campus (IC-TUMS) Tehran, Iran; 2 Immunology, Asthma & Allergy Research Institute (IAARI), Tehran University of Medical Sciences, Tehran, Iran; 3 Department of Immunology, School of Public Health, Tehran University of Medical Sciences, Tehran, Iran

**Keywords:** Epitopes, Der p 23, Allergen Immunotherapy, House Dust Mite, immunoinformatics

## Abstract

House dust mite (HDM) allergy is the leading cause of IgE-mediated hypersensitivity. Therefore identifying potential epitopes in the Dermatophagoide pteronyssinus 23 (Der p 23), a major house dust mite allergen will aid in the development of therapeutic vaccines and diagnostic kits for HDM allergy. Experimental methods of epitope discovery have been widely exploited for the mapping of potential allergens. This study sought to use immunoinformatic methods to analyze the structure of Der p 23 for potential immunoreactive B and T-cell epitopes that could be useful for AIT and allergy diagnosis. We retrieved a Der p 23 allergen sequence from Genbank database and then analyzed it using a combination of web-based sequence analysis tools including the Immune Epitope Database (IEDB), Protparam, BCPREDS, ABCpred, BepiPred, Bcepred among others to predict the physiochemical properties and epitope spectra of the Der p 23 allergen. We then built 3D models of the predicted B-cell epitopes, T cell epitopes and Der p 23 for sequence structure homology analysis. Our results identified peptides 'TRWNEDE', 'TVHPTTTEQPDDK', and 'NDDDPTT' as immunogenic linear B-cell epitopes while 'CPSRFGYFADPKDPH' and 'CPGNTRWNEDEETCT' were found to be the most suitable T-cell epitopes that interacted well with a large number of MHC II alleles. Both epitopes had high population coverage as well as showing a 100% conservancy. These five Der p 23 epitopes are useful for AIT vaccines and HDM allergy diagnosis development.

## Background

House dust mites (Dermatophagoides pteronyssinus (Der p)) are among the most important etiologic agents of IgE-mediated allergy. At least 20 Der p allergens from HDM have been identified, with De p 1, Der p 2 and Der p 11 being classified as major allergens (showing sensitization in more than 50% of patients) [1]. In atopic individuals, HDM IgE-mediated allergic reactions occur after a sensitized patient comes in contact with one or more HDM groups of allergens, resulting in an overproduction of Der p-specific IgE antibodies. The symptoms of IgE-mediated diseases range from mild allergic rhinitis, dermatitis, conjunctivitis, sometimes to life threatening anaphylaxis and allergic asthma [2]. It has been demonstrated that allergen immunotherapy (AIT) is the only effective way of treatment that addresses the underlying mechanisms of IgEmediated reactions. AIT is based on the repeated administration of disease causing allergens over a long period of time with the primary aim of establishing long-term clinical tolerance to allergens [3]. AIT has been performed using crude allergen extracts since its inception. However, AIT with whole allergen extracts has been associated with side effects due to the composition of extracts since they are usually a complex mixture of proteins [4]. Hence to find new remedial alternatives, AIT development strategies are now centered on identifying epitopes responsible for allergic responses and designing of appropriate hypoallergenic AIT vaccines [5].

Recently Der p 23 has also been characterized and classified as a major HDM allergen that reacts with 74% of patients' IgE antibodies [6]. Since the discovery of Der p 23, attempts have been made to come up with its hypoallergenic derivatives for AIT [7]. However, there has been no report of Der p 23's full B and Tcell epitope spectra so far. Therefore, the present study sought to analyze Der p 23 protein sequences as well as to identify its potentially immunogenic B and T-cell epitopes using in bioinformatics. The main objective of epitope prediction for AIT is to design and come up with hypoallergenic molecules that can replace crude allergen extracts. Therefore, the findings of this study may prove their value through aiding devising new therapeutic modalities for immunotherapy of HDM allergy and diagnosis [5].

## Methodology

### Sequence retrieval

The amino acid sequence of Der p 23 was retrieved from the National Center for Biotechnology Information (NCBI) protein sequence database (accession no. ACB46292). For the purposes of this analysis, the signal peptide sequence (amino acid number 1- 21) was removed. The sequence was saved in FASTA format for further analysis.

### Physiochemical and secondary analysis

ProtParam tool was used to analyze the physiochemical properties of the Der p 23 protein sequences [8]. The parameters analyzed included molecular weight, theoretical isoelectric point (pI), amino acid composition, total number of negatively charged residues (Asp + Glu), instability index, aliphatic index, total number of positively charged residues (Arg + Lys), net charge and the grand average of hydropathicity (GRAVY) of Der p 23. VADAR server was used to analyze the secondary and 3D structures of the Der p 23 allergen [9].

### Solvent accessibility surface (SAS) analysis

SAS analysis is commonly used to evaluate how exposed or buried a given amino acid residue is buried within a protein. The SAS of Der p 23 structure was computed using Surface Racer Program with probe radius of 1.4Å [24]. The SAS data were used in the subsequent B-cell epitope screening.

### Prediction of B-cell epitopes

Four immunoinformatics tools namely BCPREDS, ABCpred, BepiPred and Bcepred were used to predicate the B-cell epitopes from a whole sequence of Der p 23 using their default threshold values [10, 11, 12, 13]. Results of the four prediction tools were aligned together and overlapping sequences were assumed to be B-cell epitopes.

### Confirmation of surface accessibility, hydrophilicity, flexibility and antigenicity for predicted epitopes

B-cell epitopes are characterized by four parameters namely surface accessibility, antigenicity, flexibility and hydrophilicity. Therefore, Karplus and Schulz flexibility prediction (threshold: 1.000), Emini surface accessibility prediction (threshold: 1.000), Parker hydrophilicity prediction (threshold: 3.000), Kolaskar and Tongaonkar antigenicity prediction (threshold: 1.000) were applied to further screen for the most appropriate B-cell epitopes from the sequences initially predicted by BCPREDS, ABCpred, BepiPred and Bcepred [14]. The results obtained were verified using Vaxijen v 2.0 server with 0.5 taken as the threshold [15].

### Prediction of T-cell Epitopes

T-cell epitopes are predicted indirectly by identifying the binding of peptide fragments to the MHC complexes. This is achieved by estimating the strength of a peptide binding to MHC complex at a set threshold. In this study, MHC II HLA-DQA1*01:01/ DQB1*05:01, HLA-DRB1*11:01, HLA-DRB1*03:01 and HLADRB1* 15:01 restricted T-cell epitopes were predicted using the online prediction applications, the Immune Epitope Database (IEDB) 3.0 [14]. This tool represents the probability of a particular amino acid sequence forming a T-cell epitope by assigning a score. The higher the assigned score to a particular amino acid sequence, the greater the probability of that region forming antigenic epitopes [14].

### Analysis of population coverage and allergenicity of the predicted epitopes

For an epitope to be considered a good vaccine candidate, it should effectively cover human population. Thus in order to determine the population coverage of the predicted Der p 23 epitopes, T-cell epitope sequences with the corresponding HLA alleles were submitted to the population coverage analysis tool of IEDB by maintaining the default analysis parameters [14].

### Homology modeling and validation of Der p 23 epitopes

Homology modeling is the prediction of a 3D structure of a given protein to its atomic resolution based on its primary sequence. The template was modeled based on a partial crystal structure of Der p 23 using the academic version of MODELLER9v4 [23] and evaluated using GA341 and Discrete Optimized Protein Energy (DOPE) assessment functions of MODELLER. Stereochemical quality of the generated model was evaluated using ERRAT validation tools [16]. On the other hand, 3D models of predicted B and T-epitopes were constructed using PEP-FOLD [25]. After verifying them for errors using ERRAT [16], they were then superimposed on the modeled 3D structure of Der p 23 using Pymol 3D structure visualisation software (http://www.pymol.org/) for comparison of the generated epitope structures to the Der p 23 model.

## Results

### Physiochemical and secondary structure analysis

**Figure 1:**
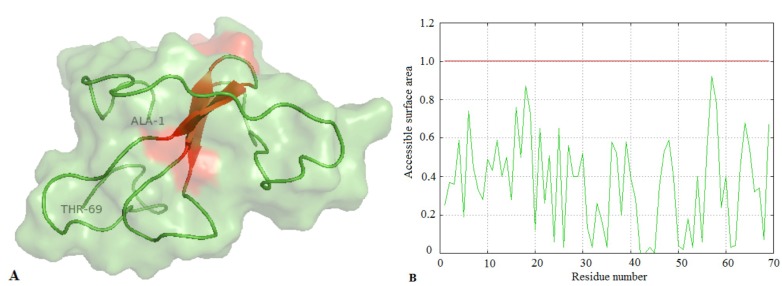
Predicted 3D structure and solvent accessibility area of Der p 23 sequences (A) A cartoon representation of the 3D structural model of Der p 23. It is composed of three antiparallel B-strands (red). The majority of the molecule is composed of a long chain of random coils (green) from the N-terminal (alanine) to the C-terminal (threonine). (B) Solvent accessibility area plot of Der p 23 shows that the allergen molecule is likely to be rich in charged and polar residues.

The primary sequence of Der p 23 used for this analysis contained 69 amino acids. It had a molecular weight of 7981.46. The theoretical pI was 4.32 indicating that it is an acidic protein. The GRAVY was -1.391 meaning that Der p 23 exhibited hydrophilic character. Table 1 Der p 23 was designated as unstable with a value of 60.52 (cut off point 40) [8]. The aliphatic index of Der p 23 was 18.41 and its estimated half-life predicted in mammalian, yeast and Escherichia coli cells was 4.4 h, > 20 h, > 10 h, respectively (Data not shown in Table 1). In addition, Der p 23 was shown to be an unfolded protein lacking helices. The majority of residues, (80%) in the sequence were located within the region of random coils Figure 1.

### Identification of B-cell epitopes

Table 2 shows preliminary linear B-epitopes predicted by BCPREDS, ABCpred, BepiPred and Bcepred.

### Confirmation of predicted B-cell epitopes

**Figure 2:**
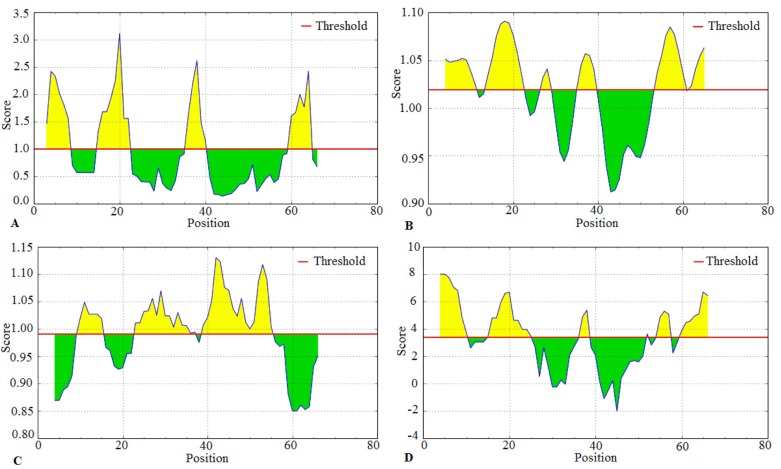
Confirmation of predicted B-cell epitopes by employing four IEDB prediction algorithms. Diagrams (A) show Emini surface accessibility scale (threshold: 1.000), (B) Karplus and Schulz flexibility prediction scale (threshold 1.000), (C) Kolaskar and Tongaonkar antigenicity scale (threshold: 1000) and (D) (Parker hydrophilicity prediction scale (threshold: 3.000). The x-axis shows amino acid residues' position in the Der p 23 sequence while the y-axis shows the correspondent score for each amino acid residue. The larger computed score for the residues is interpreted as that the residue might have a higher probability to be part of an epitope (those residues are shown in yellow in the graphs and green regions show hydrophobic regions that are highly unlikely to be part of an epitope).

Further screening of the initially predicted B-epitopes shown in Table 2 was performed taking surface accessibility, antigenicity, flexibility, and hydrophilicity of the whole Der p 23 sequence into consideration (Figure 2). Epitopes predicted by ABCpred did not show any significance in terms of these characteristics. As a result, all the seven epitopes predicted by ABCpred were discarded. Three linear B-cell epitopes, 'TVHPTTTEQPDDK','NDDDPTT' and 'TRWNEDE', which we designated B1, B2 and B3 respectively were common in BCPREDS, BepiPred and Bcepred prediction tools. They also fulfilled the key B-epitope criteria of surface accessibility, hydrophilicity flexibility and betaturn (Figure 2). Our results also showed that more than half of the total residues lying in each predicted final B-cell epitopes were hydrophilic (B1=77%; B2=86%; and B3=86%). All B-cell epitopes were located on the surface of the Der p 23 molecules and Threonine (T) was most common residue in the three epitopes constituting at least 14% of residues in each epitope. Based on their VaxiJen scores, the three peptides epitopes B1, B2 and B3 were found to be antigenic (VaxiJen score ≥ 0.5) [15] and assumed to be real epitopes (Table 3).

### Identification of T-cell epitopes

The final T-epitopes restricted on regions of HLADQ* 01:01/DQB1*05:01, HLA-DRB1*11:01, HLA-DRB1*03:01, HLA-DRB1*15:01 are shown in Table 4. It was found out that epitopes 'CPGNTRWNEDEETCT' and 'CPSRFGYFADPKDPH' both comprised of 15 residues, which we depicted as T1 and T2 respectively stood out as the best potential T-cell epitope from the Der p 23 protein (Table 4). Both epitopes exhibited a 100 % conservancy [14].

## Discussion

HDM allergens play an important role in etiology of allergic diseases. Experimental methods used for characterizing epitopes are often time consuming and costly. Hence computational methods have become useful alternatives approaches for predicting and analysing epitopes from immunologically relevant allergens, saving the expense of synthetic peptides and working time [17]. Within the past decade, a number of algorithms have been developed to predict T and B-cell epitopes on protein sequences based on propensity values of amino acid properties. In the present study, we predicted T and linear B-cell epitope of Der p 23 (a major allergen) using web based bioinformatics tools. By using different tools, peptides B1, B2, and B3 were identified as immunogenic linear B-cell epitopes that fulfilled all the criteria of surface accessibility, hydrophilicity, flexibility and antigenicity [14]. On the other hand, T-cell epitopes were predicted indirectly based on the probability of MHCpeptide ligand formation, hence T1 and T2 were identified as the best T-epitopes that interacted with HLA- DQ*01:01/DQB1*05:01, HLA-DRB1*11:01, HLA-DRB1*03:01 and HLA-DRB1*15:01 [18]. The MHC-epitope peptide interaction was found to have a 100% conservancy as reported in a similar study [19].

Secondary and physiochemical analyses performed on der p 23 sequence in this study revealed that it is an acidic and unstable protein. The predicted secondary structural components of Der p 23 in this study had some differences in terms of helices and sheet composition with those reported from previous investigations of der p 23 using laboratory methods [6, 20]. However, all the predicted epitopes in this study were located within the regions of random coils of Der p 23. This is in agreement with the proposition that coils are mainly found in the regions of proteins where surfaces are exposed making it highly likely for the predicted linear B-epitopes to be real epitopes [21].

This is the first study attempting to predict Der p 23 (a major allergen) epitopes by using computational methods therefore the results may have immense immunological value for development of AIT vaccines against HDM as well as for the development of diagnostic kits targeting der p 23. However, one of the limitations of computational approaches is that the performance of prediction tools often depends on the quality of the databases. These databases are always evolving as new information is submitted. Thus, it is likely that epitope prediction results obtained for the same set of protein under the same parameters at later time may give varying results [22]. Thus, the findings of this study may need to be validated by evaluating identified epitopes in vitro. Also as a way of reducing the rate of false positives, there is need to use a larger amount of prediction tools and fine-tuning of parameters for greater accuracy of results.

## Conclusion

Identification of epitopes in Der p 23 is critical for vaccine discovery and development. Here, we report predicted B-cell/ Tcell epitopes in Der p 23. These peptides should be tested further for immunoreactivity using in-vivo analysis.

## Conflict of interest

The authors declare no conflicts of interest.

## Figures and Tables

**Table 1:** Computed physiochemical properties of Der p 23

Parameter	Value
Amino acid residues	69
Formula	C343H491N93O121S4
Molecular weight	7981.46
Total number of atoms	1052
Theoretical pI*	4.32
Aliphatic index	18.41
Asp + Glu	16
Arg + Lys	6
Instability index	60.52 (unstable)
GRAVY**	-1.391

GRAVY**- Grand average of hydropathicity, pI*- isoelectric point.	

**Table 2:** Identification of linear B-cell epitopes sequences for Der p 23 allergen

Predication tool	Predicted epitope sequences	Position	Amino residues
BCPREDS	AVHKDCPGNTRWNEDEETCT	70-90	20
	NDDDPTTTVHPTTTEQPDDK	25-44	20
	RFGYFADPKDPHKFYICSNW	50-69	20
ABCpred	KFYICSNWEAVHKDCP	62-77	16
	DDKFECPSRFGYFADP	42-57	16
	NWEAVHKDCPGNTRWN	68-83	16
	TVHPTTTEQPDDKFEC	32-47	16
	DDDPTTTVHPTTTEQP	26-41	16
	DCPGNTRWNEDEETCT	75-90	16
	GYFADPKDPHKFYICS	52-67	16
BepiPred	ANDNDDDPTTTVHPTTTEQPDDKFE	22-46	25
	DPKD	56-59	4
	PGNTRWNEDEETC	56-68	13
Bcepred	ANDND	22-26	5
	TTTEQPD	36-42	7
	TRWNEDE	80-86	7

**Table 3:** Final B-cell epitope selected

Predicted B-epitope	Position	Vaxijen score (Threshold >0.5)
TRWNEDE	80-86	0.5917
TVHPTTTEQPDDK	32-44	0.5937
NDDDPTT	25-31	1.5881

Final B-cell epitopes selected and their ranking on the Vaxijen v.2.0 scores		

**Table 4:** Final T-cell epitope selected

Epitope	Interacting MHC-II	Position	Amino acid residues	Epitope conservancy
CPSRFGYFADPKDPH	HLA-DQB1*05:01	47-61	15	100%
	HLA-DRB1*11:01			
CPGNTRWNEDEETCT	HLA-DRB1*03:01	76-90	15	100%
	HLA-DRB1*15:01			

**Figure 3:**
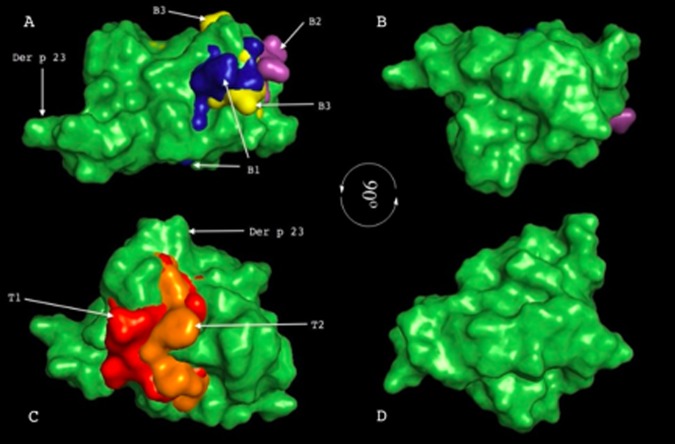
Superimposition of B and T-cell epitopes on Der p 23-allergen structure. Cartoon representation of Der p 23 rotated at 90° around the x-axis looking from the N-terminal side of the model, A and B showing the localization of three B-cell epitopes B1, B2 and B3 (blue, purple and yellow respectively) and C; D showing two T-cell epitopes, T1 and T2 (red and orange respectively) to determine their relative positions on Der p 23 allergen. It was shown that the mapped epitopes were situated closer to each other to form a single patch of epitopes on the surface of Der p 23 the protein. All the T-cell epitopes were integrated by the final part of two α-helices and their corresponding flanking loops. Overall, the three B-cell epitopes were located in the top tower regions and exposed on the outside surface and also integrated by the final part of one or two of their flanking loop.

## References

[1] Arlian LG, Platts-Mills TA (2001). J Allergy Clin Immunol.

[2] Hansen I (2004). Curr Opin Allergy Clin Immunol.

[3] Cappella A, Durham SR (2012). Hum Vaccin Immunother.

[4] Linhart B, Valenta R (2012). Curr Opin Immunol.

[5] Reisacher WR, Wang A (2013). Curr Otorhinolaryngol Rep.

[6] Weghofer M (2013). J Immunol.

[7] Banerjee S (2014). J Immunol.

[8] Gasteiger E (2003). Nucleic Acids Res.

[9] Leigh W (2003). Nucleic Acids Res.

[10] Zhang W (2012). PLoS ONE.

[11] Saha S, Raghava GP (2006). Artif Immune Syst.

[12] Larsen JE (2006). Immunome Res.

[13] Saha s, Raghava GP (2004). Artif Immune Syst.

[14] Vita R (2015). Nucleic Acids Res.

[15] Doytchinova IA, Flower DR (2007). BMC Bioinformatics.

[16] Colovos C, Yeates T (1993). Protein Sci.

[17] Saha S, Raghava GP (2006). Proteins.

[18] Yang X, Yu X (2009). Rev Med Virol.

[19] Yasmin T (2016). In Silico Pharmacol.

[20] Soh WT (2015). Int Arch Allerg Immunol.

[21] Sikic K (2010). Open Biochem J.

[22] Kulkarni A (2013). Comput Biol Chem.

[23] Sanchez R (2000). Methods Mol Bio.

[24] Tsodikov O (2002). J Comput Chem.

[25] Shen Y (2014). J Chem Theor Comput.

